# Sleep Restriction and Recurrent Circadian Disruption Differentially Affects Blood Pressure, Sodium Retention, and Aldosterone Secretion

**DOI:** 10.3389/fphys.2022.914497

**Published:** 2022-07-08

**Authors:** Ciaran J. McMullan, Andrew W. McHill, Joseph T. Hull, Wei Wang, John P. Forman, Elizabeth B. Klerman

**Affiliations:** ^1^ Renal Division, Department of Medicine, Brigham and Women’s Hospital, Boston, MA, United States; ^2^ Channing Division of Network Medicine, Brigham and Women’s Hospital, Boston, MA, United States; ^3^ Division of Sleep and Circadian Disorders, Brigham and Women’s Hospital, Boston, MA, United States; ^4^ Division of Sleep Medicine, Harvard Medical School, Boston, MA, United States; ^5^ Sleep, Chronobiology, and Health Laboratory, School of Nursing, Oregon Health & Science University, Portland, OR, United States; ^6^ Department of Neurology, Massachusetts General Hospital, Boston, MA, United States

**Keywords:** sleep timing, circadian rhythms, shift-work, sleep restriction, circadian misalignment

## Abstract

Prolonged exposure to chronic sleep restriction (CSR) and shiftwork are both associated with incident hypertension and cardiovascular disease. We hypothesized that the combination of CSR and shiftwork’s rotating sleep schedule (causing recurrent circadian disruption, RCD) would increase blood pressure, renal sodium retention, potassium excretion, and aldosterone excretion. Seventeen healthy participants were studied during a 32-day inpatient protocol that included 20-h “days” with associated scheduled sleep/wake and eating behaviors. Participants were randomly assigned to restricted (1:3.3 sleep:wake, CSR group) or standard (1:2 sleep:wake, Control group) ratios of sleep:wake duration. Systolic blood pressure during circadian misalignment was ∼6% higher in CSR conditions. Renal sodium and potassium excretion showed robust circadian patterns; potassium excretion also displayed some influence of the scheduled behaviors (sleep/wake, fasting during sleep so made parallel fasting/feeding). In contrast, the timing of renal aldosterone excretion was affected predominately by scheduled behaviors. Per 20-h “day,” total sodium excretion increased, and total potassium excretion decreased during RCD without a change in total aldosterone excretion. Lastly, a reduced total renal sodium excretion was found despite constant oral sodium consumption and total aldosterone excretion, suggesting a positive total body sodium balance independent of aldosterone excretion. These findings may provide mechanistic insight into the observed adverse cardiovascular and renal effects of shiftwork.

## Introduction

In the developed world, changes to lifestyle and work practices has diminished the amount of sleep many people obtain and altered the times workers are required to be awake (EU, 2012; Tsc et al., 2014). Acutely changing an individual’s pattern of sleep and waking times results in an abrupt shift in the timing of physical and metabolic demands on multiple physiological systems. This shift causes misalignment in timing of these actual physical and metabolic demands with the body’s anticipated demands, which are regulated by genetically encoded endogenous circadian pacemakers in the brain and in the periphery. This misalignment, between environmentally-imposed demands and endogenously anticipated demands, may lead to deleterious health effects if sustained (Bixler, 2009; Zuber et al., 2009; Vetter et al., 2016). Such effects may include alterations in brain, liver, and kidney function as well as blood pressure, all of which follow diurnal patterns resulting from the combined influences of endogenous circadian rhythmicity and exogenous activities, such as eating (Brandenberger et al., 1994; Klausen et al., 1996; Charloux et al., 1999; Voogel et al., 2001; Rejnmark et al., 2002; Zuber et al., 2009; Nikolaeva et al., 2012; Zhang et al., 2014; Reinke and Asher, 2019). Chronic misalignment, which occurs with rotating shift work (McMenamin, 2007), may increase the risk of chronic kidney disease (CKD) (Gangwisch et al., 2013; Rakova et al., 2013; McMullan et al., 2016; Uhm et al., 2018) and cardiovascular disease (Boggild and Knutsson, 1999; Brown et al., 2009). One of the most prevalent forms of recurrent circadian disruption (RCD) is shiftwork, which arises from a societal need for work operations to occur during the nighttime hours when human circadian physiology is promoting sleep, fasting, and inactivity. Accounting for approximately 20% of the United States working population (McMenamin, 2007), overnight or rotating shift workers demonstrate up to a 40% increased risk for cardiovascular disease and up to twice the odds of CKD (Uhm et al., 2018) compared to daytime workers. The longer that an individual continues in a shift work schedule, the higher their risk for cardiovascular disease (Knutsson et al., 1986; Vetter et al., 2016); discontinuation of shift work ameliorates the risk (Vetter et al., 2016).

Mechanisms underlying the association of disruptions in sleep with kidney and cardiovascular risk are incompletely understood (Torsvall et al., 1989). A growing appreciation of the role of peripheral oscillators on modulating physiological function exists and alterations in peripheral circadian clock gene expression may result in significant perturbations detrimental to normal physiological function. For example, reduced Per1 expression in the kidney alters aldosterone levels and excretion of sodium, and Per1 knock out mice exhibit increased ET-1 response to a high salt diet and non-dipping blood pressure (Douma et al., 2020; Douma et al., 2022). BMAL1 knockout mice lose diurnal patterns in glomerular filtration and aldosterone excretion and demonstrate lower blood pressure, while CRY1 knockout mice exhibit altered corticosteroid synthesis in the adrenal cortex and develop salt-sensitive hypertension (Okamura et al., 2016; Ansermet et al., 2019; Zhang et al., 2020). Several studies suggest that changes in the timing or duration of sleep affect the renin-angiotensin-aldosterone system (RAAS), either by altering the 24-h periodicity of the RAAS (Brandenberger et al., 1994; Charloux et al., 1999; Charloux et al., 2001; Haack et al., 2013; Rakova et al., 2013) or by changing total 24-h aldosterone excretion (Charloux et al., 2001; Rakova et al., 2013). There are no studies, however, of blood pressure and renal function with a carefully controlled diet under conditions of chronic sleep restriction (CSR) (which is commonly self-imposed by many people, including those partaking in shift work) and RCD.

We, therefore, tested the hypothesis that the combination of CSR and RCD would increase blood pressure, renal sodium retention, and aldosterone excretion in a tightly controlled inpatient protocol with a controlled diet. Healthy volunteers, not on blood pressure medications, were randomly assigned to either CSR (4.7 h of sleep opportunity per 20-h “day”) or Control conditions (6.7 h of sleep opportunity per 20-h “day”) while living on an imposed 20-h ‘day’ schedule (i.e., a “forced desynchrony” (FD) protocol) over 32 days (Supplemental Figure S1). On a FD protocol, the endogenous circadian system continues at its approximately 24-h rhythmicity despite the non-24-h wake/sleep (and associated feeding/fasting, activity/rest and other behaviors) schedule; this induces RCD and allows for examination of outcomes when sleep is either in alignment (occurring during circadian “night” times) or misalignment (occurring during circadian “day” times) with the endogenous circadian system (Czeisler et al., 1999). Therefore, this protocol enabled quantifying both the independent effects of CSR and the interaction between CSR and circadian misalignment, on cardiovascular and renal physiology in healthy humans.

## Materials and Methods

### Participants

Participants were recruited using flyers, web posting, and media advertisements. All participants were healthy by history, physical exam, laboratory testing of blood and urine, psychological screening, and clinical sleep recording. None were using prescription or non-prescription medications regularly except for oral contraceptive medications. Pregnant or breast-feeding women were excluded. All participants provided written informed consent before screening. The Partners Human Research Committee approved this research, which was conducted in the Center for Clinical Investigation at Brigham and Women’s Hospital (BWH), Boston, MA. All participants gave written informed consent. The study was registered on ClinicalTrials.gov (NCT01581125). Planned primary outcome measures were reported in (McHill et al., 2018b; McHill et al., 2019).

### Experimental Protocol

Pre-inpatient study conditions–Each participant abstained from caffeine, alcohol, tobacco, over-the-counter and recreational pharmaceuticals and was asked to maintain a 10-h time-in-bed sleep opportunity per night for at least 3 weeks prior to admission. Compliance with these sleep schedules was assessed using wrist actigraphy, a sleep diary, and daily bedtime and wake time calls to a time-stamped voicemail system. During those 3 weeks and at admission, urine toxicology testing was done.

Inpatient study conditions–Each participant was studied within a time-free environment at the BWH Center for Clinical Investigation for 32 calendar days with controlled lighting conditions. The inpatient protocol began with 4 days of sleep satiation (i.e., 12-h sleep opportunities each night and had 4-h nap opportunities mid-day), followed by a baseline segment of three calendar days each with 10 h of sleep opportunity and 14 h of wakefulness. After baseline, participants entered a FD protocol in which they completed 24 cycles of a 20-h “day” schedule over 20 calendar days, advancing 4 h each cycle (days 7–27). Therefore, this protocol forced a desynchrony between the 20-h scheduled cycle of sleep/wake, fasting/feeding, posture, and other changes and the ∼24.2 h endogenous circadian cycle; this created RCD. During the 20 calendar days of FD, individuals completed four beat cycles; each beat cycle contains the complete range of timing relationships between sleep onset (20-h cycle) and circadian phase (∼24.2-h cycle). Individuals randomly assigned to CSR were scheduled 15.3 h awake and 4.7 h of sleep each 20-h “day” (equivalent to ∼5.6 h of sleep per 24-h day), while individuals assigned to the Control group were scheduled 13.3 h awake and 6.7 h of sleep each 20-h “day” (equivalent to ∼8 h of sleep per 24-h day). Participants completed the protocol with a recovery segment, consisting of five calendar days with 10-h sleep opportunities followed by 14-h wake episodes with discharge on day 32.

Determination of individual’s circadian phase–Core body temperature samples were collected at 1-min intervals using a rectal temperature sensor. These data were analyzed using non-orthogonal spectral analysis techniques (Czeisler et al., 1999) to estimate intrinsic circadian period and circadian phase for each individual (Cohen et al., 2010; McHill et al., 2018a); a circadian phase of 0° indicated the fit minimum of the circadian core body temperature rhythm.

Habitual sleep typically begins ∼270°–300° after fitted core body temperature minimum in normally entrained individuals (Dijk and Czeisler, 1994). For this analysis, “days” with scheduled sleep onset occurring between 240° and 360° of circadian phase were defined as being “circadian aligned” as this would be a time the individual would habitually sleep (approximately equivalent to sleep onset occurring between 8 p.m. and 4 a.m.), while “days” with scheduled sleep onset occurring between 60° and 180° were defined as “circadian misaligned” (McHill et al., 2018b) as this is the time an individual would habitually be awake (approximately equivalent to sleep onset occurring between 8 a.m. and 4 p.m.). This was performed separately for each participant.

Diet–Beginning the day after admission, participants received an isocaloric diet, calculated per the Harris-Benedict equation with an activity factor (1.3). Breakfast was provided at 01:25 h post-awakening, lunch at 05:30 h post-awakening, and dinner at 09:30 h post-awakening each wake episode, in both conditions. The diet consisted of 45–50% carbohydrate, 30–35% fat, 15–20% protein, 150 mEq of sodium, 100 mEq of potassium, and at least 2.5 L of water per 20-h “day.”

Intravenous fluids–Individuals received a continuous infusion of 0.45% saline at a rate of 40 ml/hour during the study, to maintain patency of the intravenous draw line, contributing 62 mEq of sodium per 20-h “day.”

Blood Pressure measurement–Blood pressure was measured on the non-dominant arm using an automated blood pressure cuff (Dinamap VC150, GE Healthcare, Milwaukee WI). Blood pressure was measured ∼1 h after awakening; participants spent that hour after awakening in a bed elevated to ∼45° in a constant posture while they performed neurobehavioral testing (McHill et al., 2018b).

Urine sampling–Urine was collected immediately after awakening, every 3–6 h when the participant was awake, and at spontaneous voids. It was aliquoted, immediately frozen, and stored. Renal aldosterone excretion was measured by a modification of an ELISA immunoassay following acidification and partition extraction (IBL America, Minneapolis, MN) with CV 16.4%. Renal potassium excretion was measured using flame photometry (Cole Parmer, Vernon Hills, IL) with CV 1%. Renal sodium excretion was measured using an ion-selective electrode in a Roche/Hitachi Cobas C System with CV 3.7%. Rates of renal aldosterone, potassium, and sodium excretion were calculated as the total amount of aldosterone (µg), potassium (mEq), or sodium (mEq) excreted in each void divided by the duration of collection (hours) and temporally referenced to the midpoint between collection of the previous void and the measured void. Then, data were analyzed by 20-h “day.” As an example for the “day“ of wake episode 14, renal aldosterone, potassium, and sodium were measured in all urine produced from the start of wake episode 14 to the end of sleep episode 14 (excluding first urination of wake episode 14 and including first urination on wake episode 15) and blood pressure measured at the start of wake episode 15 (Supplemental Figure S1).

### Statistics

Data points (e.g., time of sleep episode, time of urine void) were assigned a circadian phase, binned for each participant in 60° circadian phase bins, and then referenced to the center of each bin. Blood pressure and heart rate data were analyzed using mixed-effects models with the condition and circadian phase as fixed effects and participant as a random effect to account for interparticipant differences.

Renal sodium, potassium, and aldosterone excretion were first log-transformed and then analyzed using mixed-effects models with the circadian phase or length of time awake as fixed effects and the participant as a random effect separately for both aligned and misaligned days. For each individual, the total renal aldosterone, potassium, and sodium excreted over a 20-h “day” and paired T-tests were used to compare differences during alignment and misalignment.

To test for the impact of prolonged RCD with or without CSR, paired T-tests were used to compare blood pressure, and total renal excretion of sodium, potassium, and aldosterone in Beat cycle 1 vs. Beat cycle 4. Pearson correlations were used to test for relationships between changes in renal sodium, potassium, and aldosterone and changes in blood pressure between beat cycles one and 4. As individual analysis assessed independent hypothesis, no adjustment for formal multiple testing was applied.

In sensitivity analyses, each participant’s sodium consumption, via either diet or IV infusion, was reviewed for deviation from study protocol. Individuals who lost IV access for any amount of time were identified. If these individuals received an irregular amount of half-normal saline infusion during intervals of alignment as compared to misalignment, they were excluded from analysis comparing renal sodium and aldosterone excretion during alignment and misalignment. Two participants met this criterion.

## Results

Seventeen healthy, nonsmoking, drug- and medication-free (except for oral contraceptives) adults (10 women, seven men) with a mean ± standard deviation age of 26.1 ± 4.5 (range 20–34) years completed this study (McHill et al., 2018b). The study population had a body mass index of 24.0 ± 3.6 (range 18.2–28.4) kg/m^2^ and a mean resting systolic blood pressure of 109 ± 11 (range 90–126) mmHg.

### Blood Pressure and Heart Rate

**FIGURE 1:**
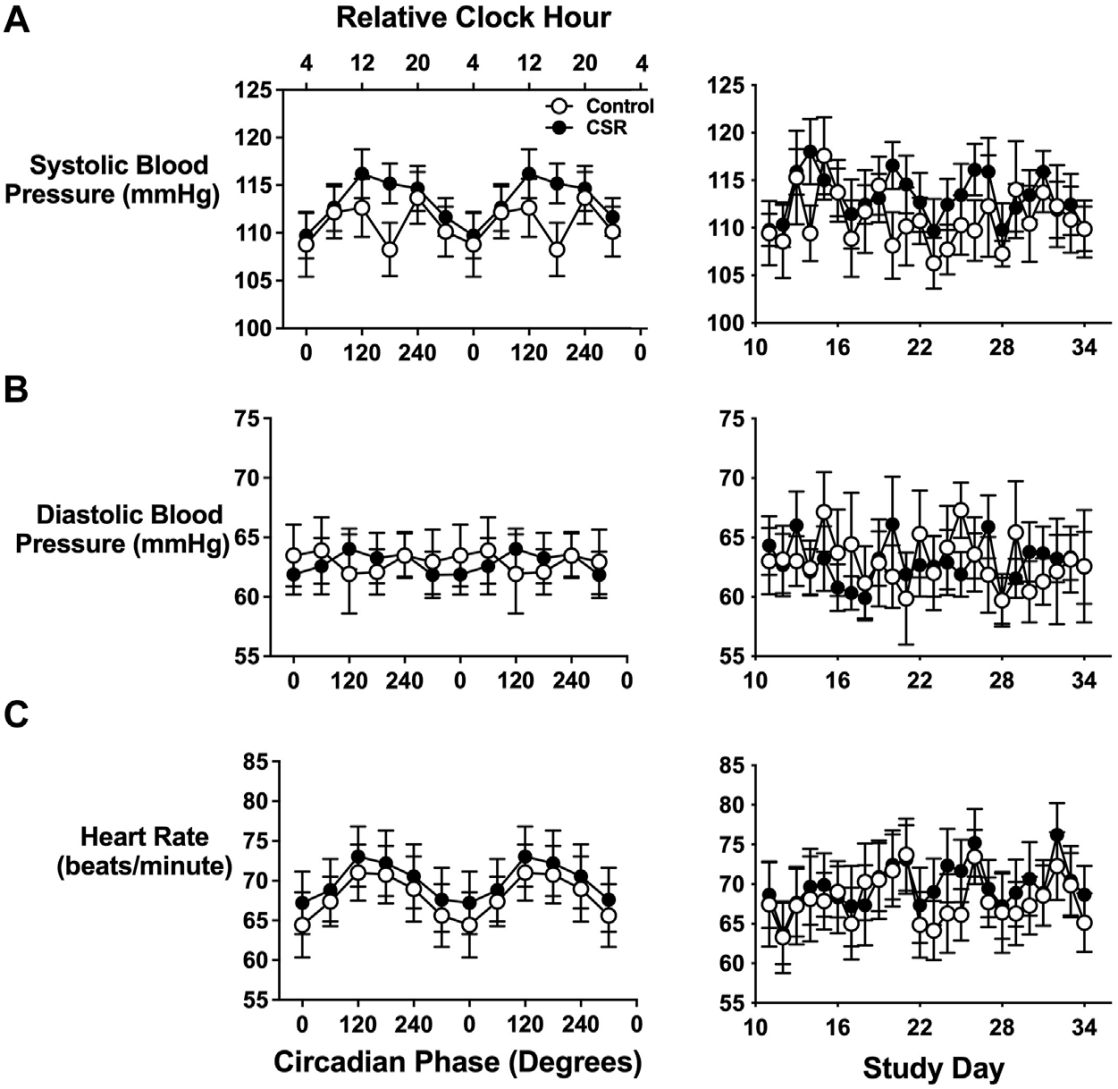
Effects of circadian phase (i.e., timing) and sleep duration on first-awake. **(A)** Systolic and **(B)** diastolic blood pressure and **(C)** heart rate. Data presented in the left column are displayed by circadian phase and the right column by wake episode day during the forced-desynchrony portion of the protocol. Vitals are displayed as mean (SEM) with respect to circadian phase (calculated from core body temperature) for the groups assigned to Control (*n* = 7) and Chronic Sleep Restriction (CSR) conditions (*n* = 9). Time of day along the top *x*-axis is plotted as relative clock hour with core body temperature minimum arbitrarily assigned a value of 04:00 h and all other times referenced to this value. Data were analyzed using analyzed using mixed-effects models with condition and circadian phase as fixed effects and participant as a random effect.

Blood pressure and heart rate were measured approximately 1 h after awakening while the individual was still in a semi-recumbent posture. Systolic blood pressure displayed a clear circadian periodicity (*p* < 0.0001) with a significant interaction between alignment (defined as scheduled sleep onset occurring at circadian phases 240° to 360°)/misalignment (defined as scheduled sleep onset occurring at circadian phases 60° to 180°) and CSR versus Control condition (*p* = 0.02)) (Figure 1A). The morning after misaligned sleep, individuals assigned to CSR demonstrated an 8.4 mmHg higher systolic blood pressure compared with those in the Control group (*p* = 0.04 for awakening at 180°, equivalent to 16:00). Awakening diastolic blood pressure did not significantly vary by alignment/misalignment (*p* = 0.85) or CSR/Control conditions (*p* = 0.43) (Figure 1B). Heart rate displayed clear circadian periodicity (*p* < 0.0001), but it did not differ between CSR/Control conditions (*p* = 0.96) (Figure 1C).

### Circadian Phase and Time Awake of Renal Sodium, Potassium and Aldosterone Excretion

Urine was collected immediately after awakening, every 3–6 h when the participant was awake, and at spontaneous voids. Rates of renal excretion were calculated as the total amount of a metric (e.g., sodium) excreted in each void divided by the duration of collection (hours) and temporally referenced to the midpoint between collection of the previous void and the measured void.

**FIGURE 2:**
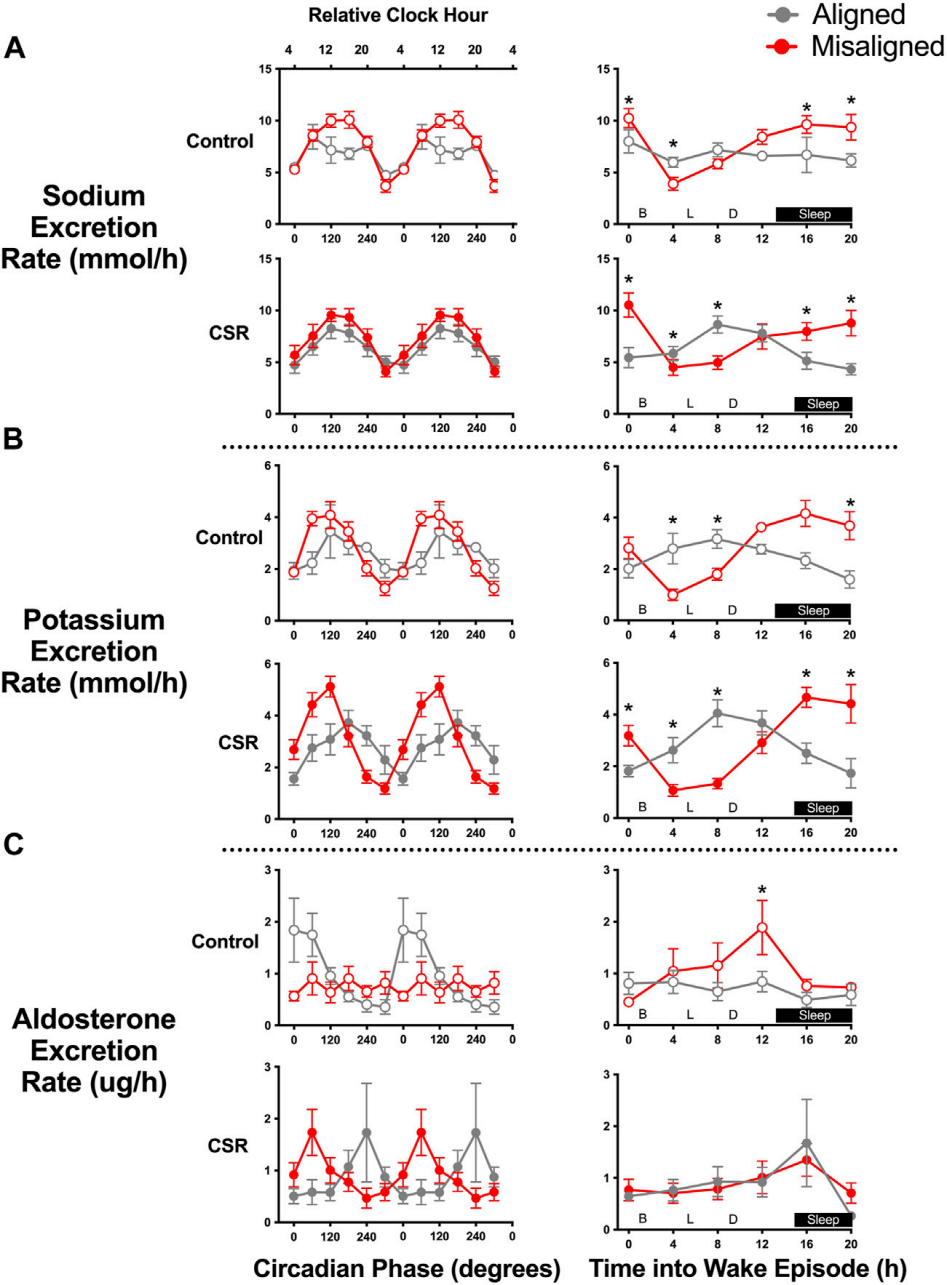
Effect of alignment and misalignment of sleep/wake cycle to night and day on **(A)** renal sodium, **(B)** potassium, and **(C)** aldosterone excretion. Left panels: Mean (SEM) rate of sodium, potassium, and aldosterone excretion in each void according to alignment or misalignment of the sleep/wake cycle with day and night is plotted by circadian phase for Control (*n* = 7) and Chronic Sleep Restriction (CSR) conditions (*n* = 9). Right panels: Mean (SEM) rate of sodium, potassium and aldosterone excretion in each void for participants during aligned and misalignment of sleep/wake cycle with day and night is plotted by time into wake episode for Control and CSR conditions. Time of day along top *x*-axis is plotted as relative clock hour with core body temperature minimum arbitrarily assigned a value of 04:00 h and all other times referenced to this value. Data were analyzed using analyzed using mixed-effects models with condition and circadian phase as fixed effects and participant as a random effect. * denotes significant difference between data points using independent t-tests after Bonferroni correction (*p* ≤ 0.008). B denotes breakfast provided at 01:25 h post-awakening, L denotes lunch at 05:30 h post-awakening, and D denotes dinner at 09:30 h post-awakening.

The rate of renal sodium excretion demonstrated a circadian rhythm under both CSR and Control conditions, with a 2–3-fold change in rate of sodium excretion between trough and peak (both *p* < 0.0001) (Figure 2A). The circadian patterns of renal sodium excretion were similar for individuals under CSR and Control conditions (*p* = 0.45) and were preserved during alignment and misalignment (*p* = 0.44). A significant alignment/misalignment by time awake effect was found for both CSR and Control conditions (both *p* < 0.01), such that sodium excretion differed at different durations of times awake depending on alignment/misalignment. Taken together, these data suggest that the temporal pattern of sodium excretion is predominantly regulated by endogenous circadian rhythms and much less determined by timing of sleep, physical activity, postural changes, or meal times.

Renal potassium excretion demonstrated a robust circadian phase effect in both CSR and Control conditions (both *p* < 0.0001) (Figure 2B). The circadian patterns of renal potassium excretion were similar for both conditions (*p* = 0.53); a slight lag, however, was found in the excretion during alignment compared with misalignment of scheduled sleep onset relative to circadian phase, with peak renal potassium excretion occurring at a later circadian phase (120°, equivalent to 12:00, vs. 180°, equivalent to 16:00) during alignment cycles (*p* = 0.005). For potassium excretion, a significant alignment/misalignment by time awake effect was found for both CSR and Control conditions (both *p* < 0.01), such that potassium excretion profiles were almost antiphase across time awake between alignment/misalignment (Figure 2B). This suggests that the temporal pattern of potassium excretion is strongly regulated by both endogenous circadian rhythms and behaviors associated with sleep/wake scheduling.

Renal aldosterone excretion displayed circadian phase effects in both CSR and Control conditions (both *p* < 0.05), with significant circadian alignment/misalignment by circadian phase interactions, such that peak circadian phase rhythms differed depending on alignment/misalignment of scheduled sleep onset with circadian phase in Control (0°, equivalent to 04:00, vs. 180°, equivalent to 16:00) and CSR (240°, equivalent to 20:00, vs. 60°, equivalent to 08:00) conditions (both *p* < 0.05). Renal aldosterone excretion did not demonstrate any significant alignment/misalignment by time awake effect in either condition (both *p* > 0.16) (Figure 2C). Taken together, these data suggest that renal aldosterone excretion was primarily dependent on behaviors associated with the sleep/wake schedule with some interaction with circadian phase.

### Analyses by 20-h “Day”

**FIGURE 3:**
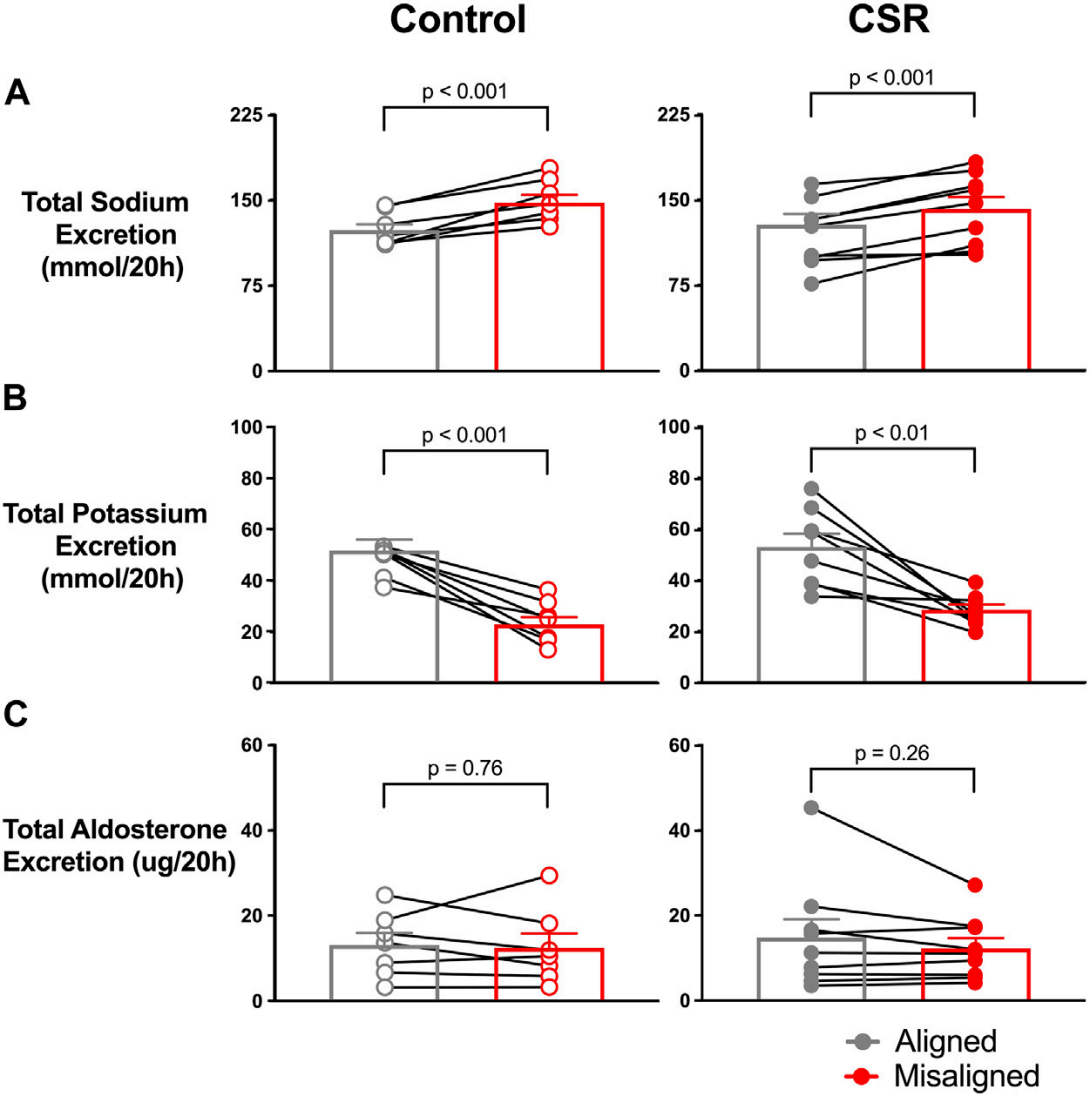
Effect of alignment (gray symbols) and misalignment (red symbols) of sleep/wake cycle to night and day on total **(A)** renal sodium, **(B)** potassium, and **(C)** aldosterone excreted over a 20-h “day” for each individual as well as the overall mean (SEM) plotted for Control (*n* = 7) and Chronic Sleep Restriction (CSR) conditions (*n* = 9). Statistical *p*-value given is from a paired *t*-test.

For analyses by 20-h “day,” all urine produced after the first void after awakening of one wake episode through the first void of the next wake episode were combined. Total renal sodium excretion during the 20-h “day” was significantly lower during alignment (121.0 ± 9.6 mEq/20 h) than during misalignment (141.8 ± 10.5 mEq/20 h, *p*-value < 0.001) (Figure 3A). This pattern was present in individuals in both CSR/Control conditions; no significant interaction occurred between alignment/misalignment and condition (*p* = 0.12). In sensitivity analyses that excluded two participants that demonstrated irregular administration of intravenous (IV) saline during the protocol due to short-term loss of IV access, the results were unchanged.

The pattern of renal potassium excretion in the study was the opposite of renal sodium. The total renal potassium excretion during a 20-h “day” was significantly higher during circadian alignment (53.0 ± 5.1 mEq/20 h) compared with misalignment (28.4 ± 1.0 mEq/20 h, *p* < 0.01) (Figure 3B) in both Control and CSR conditions.

For both conditions, mean total renal aldosterone excretion was similar during circadian alignment/misalignment (both *p* > 0.13) (Figure 3C).

### Change Between Beginning and End of RCD

**FIGURE 4:**
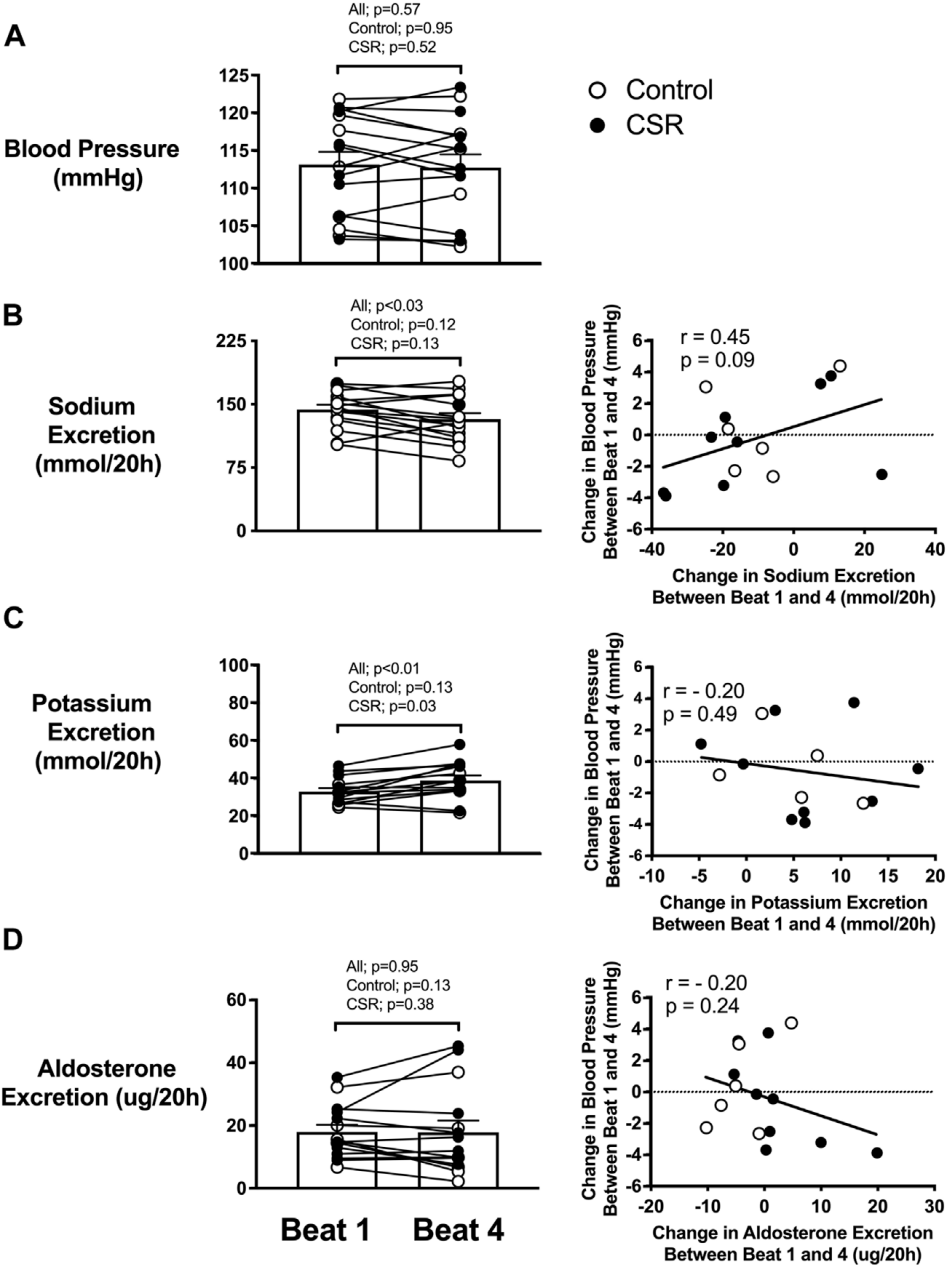
Effect of recurrent circadian disruption on blood pressure and renal excretion rates and the associations of their changes across beat cycles. Left panels: Difference between beginning (i.e., Beat cycle 1) and end (i.e., Beat cycle 4) of protocol in awakening **(A)** blood pressure, and total renal **(B)** sodium, **(C)** potassium, and **(D)** aldosterone excretion over a 20-h “day” for each individual as well as the overall mean (SEM) plotted for Control (*n* = 7) and Chronic Sleep Restriction (CSR) conditions (*n* = 9). Beat cycle is defined in main text. Statistical *p*-value given in the left columns are from paired t-tests. Right panels: Correlation between change in total sodium, potassium and aldosterone excretion with the change in blood pressure for Control and CSR conditions. The dashed line represents no change in outcomes across beat cycles, and the solid line represents the Pearson correlation regression line with the corresponding r and *p* values.

To appropriately include the complete range of relationships between sleep onset (20-h cycle) and circadian phase (∼24.2-h cycle on average (Duffy et al., 2011)) in analyses, data were analyzed related to beat cycle, with a beat defined as a contiguous sequence of days needed to encompass the full circadian cycle; for the combination of 20-h and ∼24.2-h, this equated to 6 days. Blood pressure did not significantly change across beat cycles (Figure 4A). Mean total renal sodium excretion over a 20-h “day” was significantly lower during the last beat of FD compared with the first beat cycle of RCD (Figure 4B) (*p* = 0.03). A non-significant trend was found such that the change in total sodium excretion across the protocol was positively associated with the change in blood pressure across beat cycles (r = 0.45, *p* = 0.09) (Figure 4B). Since the total content of sodium consumed each wake episode was constant throughout the study, the difference in renal sodium excretion between alignment/misalignment and as RCD continued suggests oscillating total body sodium content between alignment/misalignment with a net accumulation of total body sodium across the study in both Control and CSR conditions.

Mean total renal potassium excretion over a 20-h “day” was significantly higher during the last beat cycle of FD compared to the first beat cycle (Figure 4C) (*p* = 0.03), with no significant relationship between change in potassium excretion and change in blood pressure (r = −0.20, *p* = 0.49). Since the total content of potassium consumed each wake episode was constant throughout the study, the increase in renal potassium excretion as RCD persists suggest a net total body potassium depletion across the study in both Control and CSR conditions.

Mean aldosterone excretion over a 20-h “day” was not significantly different from the first to last beat cycle of FD for participants in either condition (*p* = 0.95) (Figure 4D). No significant association was found between any changes in aldosterone excretion and blood pressure (r = -0.20, *p* = 0.24) in either condition.

## Discussion

In this study, experimental conditions that included CSR and RCD were used to replicate critical aspects of prolonged exposure to rotating shift work schedules. These conditions resulted in physiological responses in healthy volunteers that may help identify mechanisms by which RCD leads to poor cardiovascular health and premature mortality (Vyas et al., 2012; Vetter et al., 2016; Manohar et al., 2017). First, the combination of CSR with circadian misalignment was associated with higher systolic blood pressure, despite higher sodium excretion. Second, two patterns of renal sodium excretion emerged during prolonged exposure to RCD; the first pattern was as participants altered between circadian alignment and misalignment, net total body sodium fluctuated due to net retention during alignment and net excretion during misalignment. The second pattern evident was that over four cycles of RCD, a net total body sodium retention and potassium depletion occurred, without a change in aldosterone excretion. Third, the rate of renal sodium excretion displayed circadian rhythmicity independent of aldosterone excretion and the timing of behaviors (e.g., sleep/wake timing, eating, etc.). Fourth, the rate of renal potassium excretion displayed a circadian phase effect but was also dependent upon sleep/wake timing and associated behaviors.

The decline in renal sodium excretion from the start to the end of the protocol, despite constant oral sodium intake throughout, suggests the development a net positive total sodium body balance. This apparent renal retention of sodium did not appear to be associated with an increase in aldosterone excretion with RCD. Possible mechanisms for sodium retention during RCD could act through non-aldosterone mediated pathways or from increased renal sensitivity to aldosterone. In many cases, sodium retention through increased intravascular volume and other mechanisms leads to increased systemic blood pressure. However, in our healthy population studied under constraints of sodium retention, acute misalignment, or repeated RCD, no significant increase in blood pressure occurred (McMahon et al., 2013). The absence of a significant change in blood pressure from the beginning to end of the prolonged RCD exposure, despite apparent sodium retention, may be due to the relatively short duration of circadian disruption. In the case of rotating shift workers, over 5 years of schedules that include RCD was required before cardiovascular sequelae were observed (Vetter et al., 2016). Another potential reason for the lack of a blood pressure increase despite sodium retention could be blood pressure-regulatory mechanisms not measured. For example, recent studies suggested that the skin is a sodium reservoir with immune cells regulating sodium deposition and resorption via the lymphatic system, leading to speculation that sodium storage in the skin may exert homeostatic and blood pressure-regulatory control, and in cases of increased sodium storage, eventually leading to arterial hypertension (Wiig et al., 2013; Titze and Luft, 2017). This study was not designed to assess total body sodium content or the subcutaneous sodium distribution.

The study demonstrated an unexpected variation on the previously described circadian pattern of sodium excretion. Renal sodium excretion is known to demonstrate a temporal variability that persists when posture, wake status, and meal consumption are maintained in near-constant conditions for one or more circadian cycles (el-Hajj Fuleihan et al., 1997). During these “constant routine” conditions, renal sodium excretion is maximum in the afternoon and nadirs in the early morning. In this study, reversing the timing of sleep and eating (with its associated sodium consumption), so that they were misaligned with the circadian system, affected the total amount of sodium excreted during a sleep/wake cycle, without altering the timing of maximum or minimum excretion. The timing of renal excretion of sodium was entirely independent of the timing of sodium consumption. Misalignment was associated with a net negative sodium balance or storage of sodium. The mechanisms through which misalignment influences urinary sodium excretion may be hormonal, autonomic, or at the cellular levels since all demonstrate inherent circadian periodicity. Recent studies examining the effect of peripheral oscillators located in the kidney suggest that renal sodium excretion and adrenal aldosterone secretion are highly regulated by various clock genes including Per1, BMAL1, CRY1, and CRY2 with perturbations in these oscillators measurably altering diurnal and overall blood pressure levels (Okamura et al., 2016; Zhang et al., 2020; Douma et al., 2022). The effect of locally altered circadian physiology in the adrenal gland and kidney leading to changes in renal sodium excretion in animal studies supports the hypothesis that RCD and CSR could lead to changes in how peripheral oscillators regulate overall sodium excretion.

The study involved two comparisons of renal sodium and potassium excretion. The first comparison is of renal sodium and potassium excretion during alignment and misalignment of scheduled behaviors with the circadian system. The second comparison is of renal sodium and potassium excretion at the start and end of this protocol with RCD. In both comparisons, significant changes in renal sodium and potassium excretion occurred, and in both cases, the changes are in the opposite direction; 1) sodium excretion is higher during misalignment compared with alignment while potassium excretion is lower, and 2) sodium excretion decreases across the protocol (Beat cycle 1 vs Beat cycle 4) while potassium excretion increases. A mechanistic explanation for the changes in renal sodium and potassium excretion may come from considering first the influence circadian phase on the activity of Epithelial Na channels (ENaC) in the distal renal tubule on sodium and potassium regulation and second the effects of misalignment of oral sodium consumption with circadian phase on renal sodium excretion (Richards et al., 2012a; b; Richards et al., 2013).

Regulation of renal sodium and potassium excretion is tightly controlled by the action of aldosterone on ENaC channels located in the Principal Cells of the distal renal tubule (Canessa et al., 1994). ENaC in concert with renal outer medullary potassium channel (ROMK) and Na,K-ATPase channels effectively exchange sodium for potassium in the urine and allow sodium to be removed from the urine and returned to the blood at the expense of potassium excretion. ENaC activity and it’s modulation by aldosterone is dependent on signaling by circadian clock genes (Richards et al., 2012b). *In-vitro* studies demonstrated that circadian clock proteins Period 1 and Clock regulate expression of the alpha subunit of the ENaC by acting at the E-box promoter site, which is downstream of the hormone response element the promoter site for aldosterone induced expression of ENaC (Gumz et al., 2010; Richards et al., 2012a; Richards et al., 2013). Therefore, the effect of aldosterone on renal sodium and potassium excretion is not simply a function of aldosterone secretion but also affected by the timing of secretion relative to the circadian phase. Interestingly, the mean total renal aldosterone excretion over a 20-h sleep/wake cycle was similar during alignment and misalignment and during the first portion of this protocol. Therefore, total aldosterone secretion cannot explain the changes in renal sodium and potassium excretion observed in the study. Alternatively, temporal variation may occur in the kidneys’ sensitivity to aldosterone, such that the timing of a kidney’s exposure to aldosterone during the circadian cycle affects aldosterone’s potency at the level of the Principal Cell. The fact that aldosterone secretion appeared to be more influenced by scheduled behaviors than circadian timing could explain renal sodium excretion being higher and potassium excretion being lower during misalignment than alignment despite similar total aldosterone excretion in both CSR and Control conditions. Importantly, this exhibits parallels to what has been described for glycemic regulation (Morris et al., 2015); individuals secrete similar quantities of insulin in response to a standardized meal given during circadian alignment and misalignment, but during misalignment, they demonstrate decreased insulin sensitivity and therefore increased post-prandial hyperglycemia.

Despite our participants’ consuming a fixed intake of sodium and potassium during each wake episode, and contrary to expectations, significant variability occurred in individual’s total renal sodium and potassium excretion during the 20-h cycle with RCD; more sodium excretion occurred during misaligned sleep/wake cycles compared to aligned sleep/wake cycles. Furthermore, sustained RCD led to a decrease in renal sodium excretion, suggesting dietary sodium retention with total body potassium depletion. This variability in sodium balance was previously suggested in a study of individuals under long-term space flight simulation that found an infradian periodicity (approximately monthly) in sodium balance. The variability in sodium balance seen in that report was attributed in part to participants being regularly exposed to shifts of prolonged wakefulness, which led to wakefulness and consumption of food during circadian night (Rakova et al., 2013). In our study, individuals were not exposed to prolonged intervals of activity, wakefulness, or eating, but they did remain awake and consumed food during the endogenous circadian night during misalignment portions of the protocol, therefore duplicating some but not all of the circumstances of that previous study. We did not observe significant differences between CSR and Control conditions in sodium excretion, in which the CSR group was awake for 2 h longer each “day” compared with the control group, further supporting the argument that food intake at night is driving these changes as opposed to longer wakefulness.

Our observation that awakening systolic blood pressure was increased following misalignment in those with CSR is novel and supports observational evidence (Morris et al., 2017). While a circadian rhythm in blood pressure has been previously described (Shea et al., 2011), the impact of CSR in modulating the effect of circadian timing has not previously been reported. The clinical significance of this result lies in the high prevalence of short sleep duration–and therefore CSR–among individuals working night shifts who are also exposed to circadian misalignment (Cheng and Cheng, 2017; Korsiak et al., 2017). Conversely, the results could possibly be viewed as evidence that sleep is protective against increases in blood pressure that occur from misalignment; this is supported from an analysis of 9,200 individuals in the United Kingdom Biobank demonstrating that shift workers with short sleep duration were two fold more likely to require antihypertensive medications than those with long sleep (Riegel et al., 2019). Explanations for the increased blood pressure include arterial stiffness, glucocorticoid secretion, inflammation and sympathetic tone; all have been reported as higher in chronic shift workers or individuals undergoing acute misalignment (Jankowiak et al., 2016; Morris et al., 2016; Boettcher et al., 2017; Neufeld et al., 2017). In this protocol, since renal sodium excretion increased during misalignment, sodium retention cannot explain this increase in systolic blood pressure with misalignment. In fact, we observed a non-significant trend for greater sodium excretion across the study to be associated with higher blood pressure, potentially suggesting an acute healthy response to an increase in blood pressure in our healthy population. Other mechanisms, including increased sympathetic tone during instances of misalignment, might lead to the increase in systolic blood pressure found here. Short sleep duration has been reported to increase sympathetic and lower vagal tone. The combination of short sleep duration with misalignment of sleep may be additive in increasing sympathetic tone and result in the acute increases in blood pressure seen during misalignment (Castro-Diehl et al., 2016; Neufeld et al., 2017). These changes may contribute to adverse cardiac and renal outcomes seen in individuals working extended, night, and/or shift work schedules (Tsc et al., 2014; McMullan et al., 2016; Vetter et al., 2016). We note no significant differences in cortisol secretion by circadian phase between CSR and Control groups were found these participants, thereby suggesting that altered cortisol secretion acting via the aldosterone receptor is an unlikely explanation for the changes in renal sodium and blood pressure observed (McHill et al., 2018a).

Despite the rigorous protocol design and tightly controlled conditions, this study demonstrates several limitations. First, the number of participants, 17, limits the statistical power to evaluate some interactions between metrics. However, the multiple data observations during each sleep/wake cycle for each participant with heavily controlled inpatient conditions reduced inter-individual variability and increased statistical power. Second, misalignment arising from a FD protocol does not replicate all aspects of misalignment encountered by rotating shift workers. However, a FD protocol does allow the effects of misalignment to be separated from the effects of change in sleep/wake timing and associated behaviors. This feature is important in a study aiming to assess circadian physiology of renal sodium handling independent of sleep/wake timing and associated behaviors, and it avoids confounding these two conditions as has occurred in previous studies (Rakova et al., 2013). Future work should expand these findings in actual shift-work populations. Third, we assessed blood pressure at the start of each wake episode. Measuring blood pressure at several time points in each sleep/wake cycle would have provided more information on blood pressure during alignment and misalignment and the effect of repeated short sleep duration on blood pressure. Fourth, our participants were healthy with no evidence of hypertension or renal dysfunction or any medications at baseline. It is not clear how these results would apply to people with hypertension and renal dysfunction, to whom medications affecting blood pressure and renal sodium handling are commonly prescribed.

We systematically investigated the separate effects of sleep timing and duration, wake duration and associated behaviors, and circadian phase on blood pressure, renal sodium excretion, and aldosterone excretion in healthy individuals. Under CSR conditions, increased systolic blood pressure was evident during misalignment. Sustained RCD led to decreased renal sodium excretion despite minimal changes in aldosterone excretion, suggesting increased renal aldosterone sensitivity. Renal sodium excretion followed a distinct circadian pattern with little influence from timing of dietary sodium and potassium intake, behavior, or aldosterone excretion. This robust temporal circadian pattern in renal sodium excretion is consistent with *in-vitro* studies demonstrating a clear periodicity in regulation of ENaC channel transcription. This clear circadian pattern exhibits implications for clinical practice with respect to the diagnostic reliability of urine electrolyte measurements and the effect of timing of common medications that target activity of the RAAS. More basic, epidemiologic, and clinical research is needed to determine if the effects of circadian phase and misalignment are altered in shift workers in the general population and whether differences by sex, age, comorbidity (e.g., body composition, sleep apnea), diet type, or other factors exist.

## Data Availability

The raw data supporting the conclusion of this article will be made available by the authors, without undue reservation.
