# Supplementary material for: Sleep Restriction and Recurrent Circadian Disruption Differentially Affects Blood Pressure, Sodium Retention, and Aldosterone Secretion
Source: Front Physiol. 2022 Jul 8;13:914497. doi: 10.3389/fphys.2022.914497 (PMC9305384; doi:10.3389/fphys.2022.914497)
Supplement: Supplementary file 1 [file DataSheet1.docx]

Supplementary Material

## Supplementary Figures


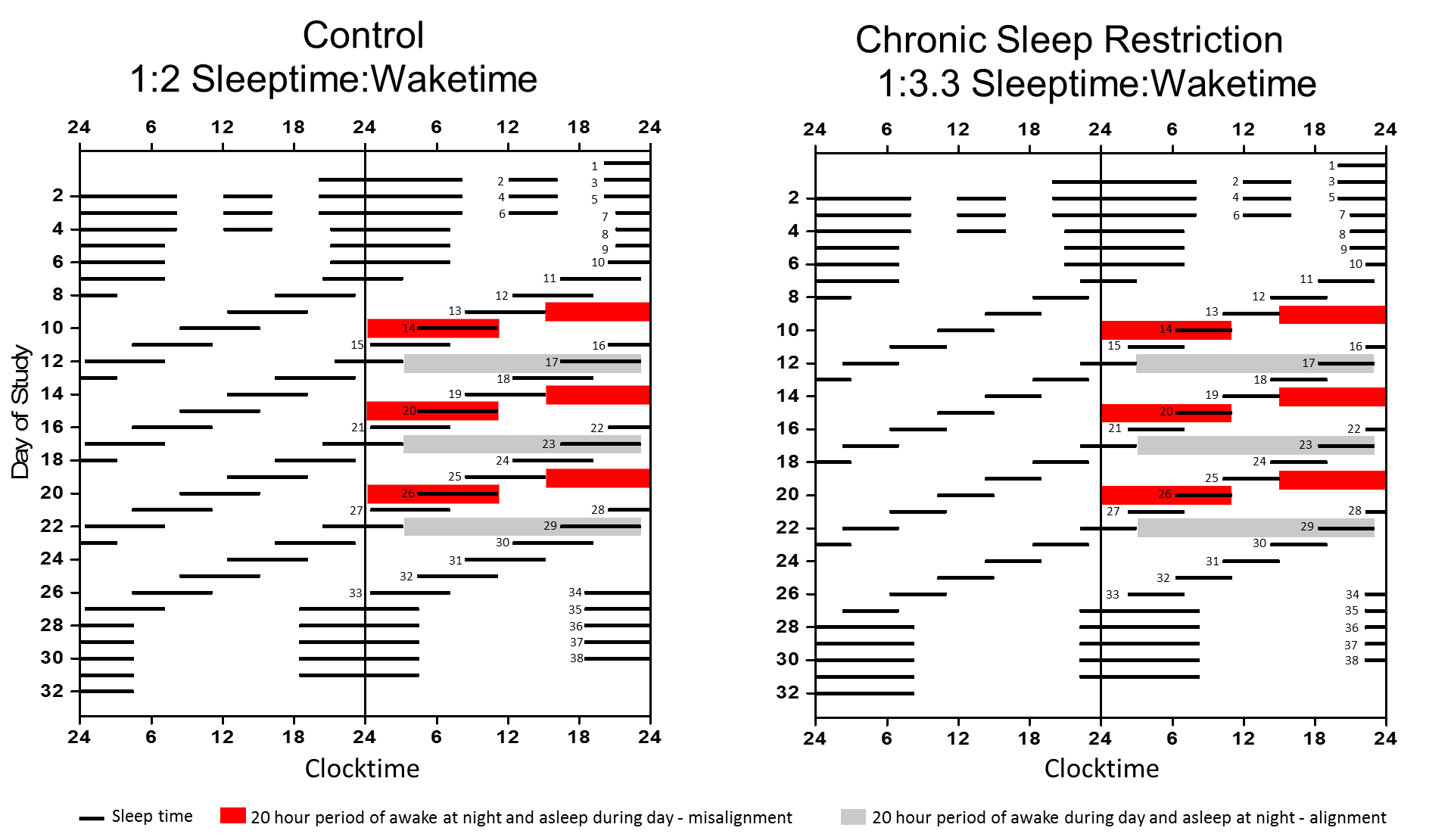


**Supplemental Fig S1:** Sleep schedule for participants during the 32-day inpatient study in double raster form. Individuals were randomized into one of two conditions; Control or Chronic Sleep Restriction (CSR). Each sleep episode is numbered. Sleep times are represented by the black line. During study days 7-27 participants lived on 20-hour ”days”. Three example 20-hour cycles during which were participants were asleep at night and awake during the day (“aligned”) are highlighted in gray and three cycles during which participants were awake overnight and slept during the day (“misaligned”) are highlighted in red).
